# Enhanced Recovery After Surgery (ERAS®) Society Consensus Guidelines for Emergency Laparotomy Part 3: Organizational Aspects and General Considerations for Management of the Emergency Laparotomy Patient

**DOI:** 10.1007/s00268-023-07039-9

**Published:** 2023-06-05

**Authors:** Carol J. Peden, Geeta Aggarwal, Robert J. Aitken, Iain D. Anderson, Angie Balfour, Nicolai Bang Foss, Zara Cooper, Jugdeep K. Dhesi, W. Brenton French, Michael C. Grant, Folke Hammarqvist, Sarah P. Hare, Joaquim M. Havens, Daniel N. Holena, Martin Hübner, Carolyn Johnston, Jeniffer S. Kim, Nicholas P. Lees, Olle Ljungqvist, Dileep N. Lobo, Shahin Mohseni, Carlos A. Ordoñez, Nial Quiney, Catherine Sharoky, Richard D. Urman, Elizabeth Wick, Christopher L. Wu, Tonia Young-Fadok, Michael J. Scott

**Affiliations:** 1 Department of Anesthesiology Keck School of Medicine, University of Southern California, 2020 Zonal Avenue IRD 322, Los Angeles, CA 90033 USA; 2 Department of Anesthesiology, Perelman School of Medicine, University of Pennsylvania, 3400 Spruce St, Philadelphia, PA 19104 USA; 3 Department of Anesthesia and Intensive Care Medicine, Royal Surrey County Hospital, Egerton Road, Guildford, Surrey, GU5 7XX UK; 4 Sir Charles Gardiner Hospital, Hospital Avenue, Nedlands, WA 6009 Australia; 5 Salford Royal NHS Foundation Trust, Stott La, Salford, M6 8HD UK; 6 University of Manchester, Manchester, UK; 7 Western General Hospital, NHS Lothian, Edinburgh, EH4 2XU Scotland; 8 Hvidovre University Hospital, Copenhagen, Denmark; 9 Center for Surgery and Public Health, Harvard Medical School, Brigham and Women’s Hospital, 1620 Tremont Street, Boston, MA 02120 USA; 10 Division of Trauma, Burns, Surgical Critical Care, and Emergency Surgery, Brigham and Women’s Hospital, 1620 Tremont Street, Boston, MA 02120 USA; 11 Perioperative Medicine for Older People Undergoing Surgery (POPS), Guy’s and St Thomas’ NHS Foundation Trust, London, UK; 12 Faculty of Life Sciences and Medicine, King’s College London, London, UK; 13 Research Department of Targeted Intervention, Division of Surgery & Interventional Science, University College London, London, UK; 14 Department of Surgery, Virginia Commonwealth University Health System, 1200 E. Broad Street, Richmond, VA 23298 USA; 15 Department of Anesthesiology and Critical Care Medicine, Department of Surgery, The Johns Hopkins University School of Medicine, 1800 Orleans Street, Baltimore, MD 21287 USA; 16 Department of Emergency and Trauma Surgery, Karolinska University Hospital, CLINTEC, Karolinska Institutet, Stockholm, Sweden; 17 Karolinska University Hospital Huddinge, Hälsovägen 3. B85, S 141 86 Stockholm, Sweden; 18 Department of Anaesthesia, Perioperative Medicine and Critical Care, Medway Maritime Hospital, Windmill Road, Gillingham, Kent, ME7 5NY UK; 19 Division of Trauma, Burns and Surgical Critical Care, Brigham and Women’s Hospital, 75 Francis Street, Boston, MA 02115 USA; 20 Division of Trauma and Acute Care Surgery, Medical College of Wisconsin, 8701 Watertown Plank Rd, Milwaukee, WI 53226 USA; 21 Department of Visceral Surgery, Lausanne University Hospital CHUV, University of Lausanne (UNIL), Rue du Bugnon 46, 1011 Lausanne, Switzerland; 22 Department of Anaesthesia, St George’s Hospital, Tooting, London, UK; 23 Kaiser Permanente Research, Department of Research & Evaluation, 100 South Los Robles Ave, 2nd Floor, Pasadena, CA 91101 USA; 24 Department of General & Colorectal Surgery, Salford Royal NHS Foundation Trust, Scott La, Salford, M6 8HD UK; 25 Department of Surgery, Faculty of Medicine and Health, School of Health and Medical Sciences, Örebro University, Örebro, Sweden; 26 Gastrointestinal Surgery, Nottingham Digestive Diseases Centre and National Institute for Health Research (NIHR) Nottingham Biomedical Research Centre, Queen’s Medical Centre, Nottingham University Hospitals and University of Nottingham, Nottingham, NG7 2UH UK; 27 MRC Versus Arthritis Centre for Musculoskeletal Ageing Research, Queen’s Medical Centre, School of Life Sciences, University of Nottingham, Nottingham, NG7 2UH UK; 28 Division of Trauma and Emergency Surgery, Department of Surgery, School of Medical Sciences, Orebro University Hospital, Orebro University, 701 85 Orebro, Sweden; 29 Division of Trauma and Acute Care Surgery, Department of Surgery, Fundación Valle del Lili, Cra 98 No. 18 – 49, 760032 Cali, Colombia; 30 Sección de Cirugía de Trauma y Emergencias, Universidad del Valle – Hospital Universitario del Valle, Cl 5 No. 36-08, 760032 Cali, Colombia; 31 Division of Traumatology, Surgical Critical Care and Emergency Surgery, University of Pennsylvania, Philadelphia, PA 19104 USA; 32 Department of Anesthesiology, The Ohio State University and Wexner Medical Center, 410 West 10th Ave, Columbus, OH 43210 USA; 33 Department of Surgery, University of California San Francisco, 513 Parnassus Ave HSW1601, San Francisco, CA 94143 USA; 34 Department of Anesthesiology, Critical Care and Pain Medicine, and Department of Anesthesiology, Weill-Cornell Medicine, Hospital for Special Surgery, 535 East 70th Street, New York, NY 10021 USA; 35 Division of Colon and Rectal Surgery, Department of Surgery, Mayo Clinic College of Medicine, Mayo Clinic Arizona, 5777 e. Mayo Blvd., Phoenix, AZ 85054 USA; 36 Department of Anesthesiology and Critical Care Medicine, and Leonard Davis Institute for Health Economics, University of Pennsylvania, 3400 Spruce St, Philadelphia, PA 19104 USA; 37 University College London, London, UK

## Abstract

**Background:**

This is Part 3 of the first consensus guidelines for optimal care of patients undergoing emergency laparotomy using an enhanced recovery after surgery (ERAS) approach. This paper addresses organizational aspects of care.

**Methods:**

Experts in management of the high-risk and emergency general surgical patient were invited to contribute by the International ERAS® Society. PubMed, Cochrane, Embase, and MEDLINE database searches were performed for ERAS elements and relevant specific topics. Studies were selected with particular attention to randomized clinical trials, systematic reviews, meta-analyses and large cohort studies, and reviewed and graded using the Grading of Recommendations, Assessment, Development and Evaluation system. Recommendations were made on the best level of evidence, or extrapolation from studies on elective patients when appropriate. A modified Delphi method was used to validate final recommendations.

**Results:**

Components of organizational aspects of care were considered. Consensus was reached after three rounds of a modified Delphi process.

**Conclusions:**

These guidelines are based on best current available evidence for organizational aspects of an ERAS® approach to patients undergoing emergency laparotomy and include discussion of less common aspects of care for the surgical patient, including end-of-life issues. These guidelines are not exhaustive but pull together evidence on important components of care for this high-risk patient population. As much of the evidence is extrapolated from elective surgery or emergency general surgery (not specifically laparotomy), many of the components need further evaluation in future studies.

## Introduction

This is Part 3 of a three-part guideline. Part 1[1] dealt with background and preoperative care including rapid assessment and diagnosis, simultaneous resuscitation and optimization, and Part 2 (Scott et al. 2023 WJS in press) covered intraoperative and postoperative care. This section covers organizational aspects of management and includes end-of-life issues. The latter important aspect of care was included as the mortality of emergency laparotomy (EL) remains high, and patients may present with low likelihood of survival [1]. Due to the diverse underlying conditions and varying presentations of this group of patients, not all pathway components will always be applicable; however, we believe the organizational aspects of care are relevant to all teams, hospitals and systems seeking to implement an enhanced recovery after surgery (ERAS) EL pathway.

## Methods

This project was initiated by the ERAS® Society. Lead authors (MS and CP) were invited by the society to establish a guideline development group (GDG) of health-care professionals with diverse clinical or academic expertise in the management of patients undergoing EL. The GDG consisted of surgeons, anesthesiologists, a nurse, a geriatrician, and a PhD who supported the organization of the literature. Several of the authors were also accredited in intensive care, and the group was selected to ensure international representation. There was equal author representation from the USA and the UK (lead authors MS and CP are both US and UK experienced), with more surgical representatives from the USA, and more anesthetic representatives from the UK reflecting National Emergency Laparotomy (NELA) audit involvement. There were five European authors and two from the rest of the world. We recognize with regret in retrospect that Asia and Africa were not included and will correct this on the next iteration of these guidelines. A list of topics was generated, and groups of physicians with different backgrounds and from different countries were assigned to each topic, based on their expertise, to perform a literature review of English language publications and then to generate recommendations using the GRADE structure [2] and a modified Delphi process. Once the topic groups had drafted recommendations, these were collated and sent to the whole group for feedback. There was then significant review, editing, and response to comments, as well as extensive discussion of appropriate inclusion or modification of the recommendation list. The paper and recommendation list were then circulated again using a modified Delphi approach to rank the strength of the recommendation and seek further comment. A final Delphi was then undertaken highlighting areas where, prior to modification, there had been less than 80% agreement, on this final round more than 80% consensus was reached. The period searched was from 2005 until September 2021, with greater emphasis on recent publications, randomized clinical trials (RCTs), systematic reviews, meta-analyses and large cohort studies. With delays in reconvening the group due to the COVID-19 pandemic, an updated search was performed in the Spring of 2022. Retrospective studies were considered where no other higher level of evidence was available, and there was particular relevance to EL. The final guidelines were then circulated to all authors for review and identification of further relevant papers. All authors had access to papers reviewed using a reference library. The guideline development process used to reach consensus on recommendations was based on the guidance published by the ERAS® Society [3, 4]. Key components of the organization of care were agreed on and assessed with three circulations of the paper. A reviewer from the International ERAS® Society (OL) was appointed to provide internal review of the guideline as it developed, on his suggestion and the need for ERAS recommendations to be measurable for compliance and actionable; the paper was re-ordered prior to the final Delhi round to place all intra and postoperative components into Part 2 (WJS in press 2023), and other components perhaps less amenable to change by clinicians, such as delivery system structure, into this paper. We would suggest that monitoring of these organizational components should occur at a hospital or system level, consider culture and context, and be separate from the clinical components in the pre-, intra- and postoperative pathways. Discussion of implementation and delivery of the whole consensus guidelines pathway is discussed in this paper.

### Definitions

In these guidelines, EL is defined in line with criteria used by large cohort studies [5, 6] as described in Part 1[1]; therefore, trauma laparotomies, appendectomy and cholecystectomy are excluded. Most vascular conditions are excluded, such as laparotomy for vascular pathology including ruptured aortic aneurysm and return to the operating room with complications following a vascular procedure. Conditions relating to bowel ischemia such as mesenteric vascular insufficiency are included. The definition of “emergency” is described in detail in Part 1[1], and in these papers (Part1-Part 3), the term “emergency” is applied to all patients with a non-elective, potentially life threatening intra-abdominal condition requiring surgery.

## Results

A summary of the ERAS elements for organizational aspects of care and general management considerations and grading of recommendations with their respective level of evidence is described in Table 1.

**Table 1:** ERAS Emergency Laparotomy Guidelines System Based Recommendations. Consensus Guideline Review and Grading using a Modified Delphi Method [2–4] Organizational Components of Care

ERAS Item Part 3 (Part 1, 2 and 3 combined)	Guideline	Level of Evidence	Recommendation Grade
	**General considerations for surgical management of the patient undergoing emergency laparotomy**		
1. (36) Organization of surgical services for delivery of emergency general surgery	Every country has a different health system structure, payment system, and geography which can either facilitate or pose barriers to optimal delivery of care. Implementing best practice will require great change across most health systems. Strong consideration should be given to establishment of surgical and perioperative care teams with expertise dedicated to the care of emergency general surgery patients to optimize outcomes	Low	Strong
2.(37) Experience of Surgeon and Anesthesiologist	Perioperative care for patients undergoing emergency general surgery and specifically EL should be allocated to surgeons, anesthesiologists and intensivists with expertise that matches the needs of the patient. Strong consideration should be given as to how such resources are made available 24 h a day	High	Strong
	**Ensuring the safe care of the emergency laparotomy patient postoperatively**		
3. (38) Postoperative Levels of Care	In an ideal health system, all patients would be admitted to ICU to have a high level of monitoring. A pragmatic approach may be required for a risk score threshold triggering admission based on local availability of ICU beds. Patients who cannot be admitted to a critical care bed, require proactive and ongoing observation, and local protocols should be developed for this situation	Moderate	Strong
4. (39) Ongoing monitoring and management of ongoing physiological derangement, early detection of complications and avoidance of failure to rescue	Local protocols should be developed to implement regular monitoring, including use of a physiological track and trigger system to alert to deterioration and complication development, to promote early intervention and prevent failure to rescue	High	Strong
	**Multidisciplinary management of the EL Patient**		
5. (40) Comprehensive Proactive Care of the Older Surgical Patient	Patients over 65 years of age should be assessed, and co-managed, as early as possible postoperatively by a physician with expertise in the care of the older surgical patient (geriatrician) and evidence-based elder-friendly practices used	Moderate	Strong
6. (41) Implications of ERAS for emergency laparotomy for nursing practice	Nurses as well as other allied health professionals are key members of an ERAS team and should be involved in all stages of the design and implementation of the ERAS pathway	Low	Strong
	Consideration should be given to the role of an emergency laparotomy program coordinator to facilitate data collection, promote ERAS pathway adherence and to provide a sense of continuity for patients as they progress through levels of care	Low	Weak
	**Measures to improve end-of-life care and reduce non-beneficial surgery**		
7. (42) Measures to improve end-of-life care and reduce non-beneficial surgery	Future emergency laparotomy studies and databases should include, when possible, a study of patients who were eligible for surgery but did not undergo surgery (the “NoLap” population) and died. Research should also explore international and cultural differences in refusal for surgery	Low	Strong
Palliative care and end-of-life management	Multidisciplinary discussions should take place before surgery where feasible for high-risk patients (high risk defined by local criteria using a validated risk scoring system including a frailty evaluation when appropriate), and after surgery on all eligible patients, as well as the “NoLap” population	Moderate	Strong
	Discussions between specialties and disciplines with patients and carers regarding benefits and risks related to surgery, and the alternatives to surgery should be clearly documented. The patients’ “Goals of Care” should be included in this documentation	Low	Strong
	Staff should have training in palliative care conversations and end-of-life management	Low	Weak
	**Audit, Pathway Implementation, and Improvement**		
8. (43) Audit	Audit is a central component of ERAS. Having a pathway with defined measurable components facilitates improvement and allows comparison of processes and outcomes between different practitioners and centers	Moderate	Strong
9. (44) Multidisciplinary Review of Outcomes	Outcomes from emergency laparotomy should be reviewed on a regular basis. Review should be multidisciplinary and multispecialty with performance compared against evidence-based standards. A structured approach with standard nomenclature and thematic analysis is essential and should be documented	Moderate	Strong
10. (45) Development and implementation of an ERAS protocol for emergency laparotomy at Health System Level	Development and implementation of an ERAS protocol for emergency laparotomy should involve patients and carers, multidisciplinary stakeholders, and senior executives. Adequate time and funding should be allocated for implementation and planning for long-term sustainability	Low	Strong
11. (46) Initial implementation of high impact components	Lessons from large scale studies suggest an implementation focus on a small number of high impact components from an ERAS pathway initially, may be more successful	Low	Weak

### Commentary

**Fig. 1:**
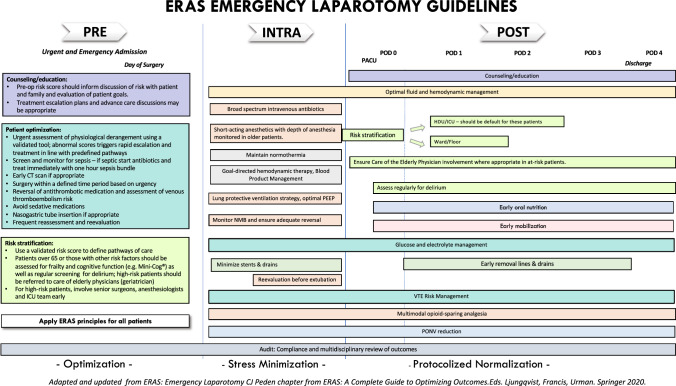
A diagrammatic representation of the whole emergency laparotomy pathway, showing specific and contiguous pre-, intra- and postoperative components. Updated and adapted from Peden CJ. Enhanced Recovery after Surgery: Emergency Laparotomy. In Enhanced Recovery After Surgery. Eds. Ljunqvist, Francis, Urman. 2020 Springer Nature, Switzerland

EL is required to treat a range of upper and lower gastrointestinal conditions in patients who also require management of acute physiological derangement before, during and after surgery. This warrants a specific EL pathway. In particular, a high level of intraoperative and postoperative monitoring is needed to ensure desired physiological parameters are attained and maintained. Many of the elements of the pathway are contiguous across pre-, intra- and postoperative phases of the pathway and are summarized in Fig. 1.

These guidelines are based on best available current evidence and will be revised when new evidence that changes the recommendations becomes available. While every effort has been made to list correct drug dosages, readers should check drug formulations and dosages in their National/Hospital formularies before prescribing.

## Organization of surgical services for delivery of emergency general surgery

Outcomes from patients undergoing emergency general surgery (EGS) and specifically EL have been shown to vary widely, with variation attributed to both patient factors and system factors [7]. Modifiable system factors include availability of intensive care beds, access to high-quality radiological services and involvement of senior health-care professionals in the delivery of all stages of care [8–12]. Improved delivery of evidence-based processes of care [13, 14] and provision of structural metrics [15] improve outcomes. Organization of services to ensure key care components delivered reliably, by highly skilled personnel, to all patients regardless of location and type of presentation, is one of the main challenges to improving care for these high-risk patients [8, 10, 15–17]*.*

Compared with standards of care for elective major surgical procedures, such as colorectal cancer, standards for EGS have been sparse. Over the last decade, awareness of organizational needs has increased [18]. Some countries have defined standards for the organization of emergency surgical services including the separation of elective and emergency surgical workload with the provision of separate teams and identification of key resources and facilities including the provision of postoperative intensive care beds [19–21]. Many health-care systems are seeing a steady yearly increase in emergency surgical cases, especially in the older population [22]. The centralization of EGS services and number of surgeries performed per annum by the surgeon has been shown to improve outcomes [23], especially in the geriatric population [22]. Different models of care have been shown to provide better outcomes for EGS, with one US study showing a 31% reduction in mortality with an acute care surgery service model compared with a general surgical service [24].

### Recommendation

Every country has a different health system structure, payment system, and geography which can either facilitate or pose barriers to optimal delivery of care. Implementing best practice will require great change across most health systems. Strong consideration should be given to the establishment of surgical and perioperative teams with expertise dedicated to the care of EGS patients to optimize outcomes.

Level of evidence: Low.

Recommendation grade: Strong.

## Experience of surgeon and anesthesiologist

Patients undergoing EL are at high risk of morbidity and mortality. There is minimal margin for error, and there is some evidence to show that outcomes improve with experienced surgical and anesthesia teams**.** A study of the American College of Surgeons (ACS) National Surgical Quality Improvement Program (NSQIP) database showed that surgery led by trainee surgeons was independently associated with increased intra- and postoperative events, wound, pulmonary, venous thromboembolic complications, and urinary tract infections [25]. However, 40% of the cases were appendectomy and 15% labeled as “other” [25]. Other studies have shown an association with the presence of a senior surgeon and anesthesiologist in the operating room and improved outcomes [14, 26, 27]. English National Health Service (NHS) organizations are now incentivized to provide a senior surgeon and senior anesthesiologist for high-risk EL cases, including all cases in frail patients, by being paid a higher tariff for such a pathway. Such an approach has been shown to be effective in improving outcomes in other emergency surgical conditions such as patients with fractured neck of femur [28]. In the USA, the ACS and the American Association for Surgery of Trauma (AAST) have recently introduced new resources and standards, including training requirements for surgeons at verified trauma/EGS centers [21].

A recent large observational study from the UK National Emergency Laparotomy Audit (NELA) database comparing trainees with consultant surgeon outcomes showed no detrimental influence on mortality or return to theater—although the level of experience of the operating surgeon was not specified [29] nor the level of supervision. However, the need for trainee exposure and management of complex pathology and emergency situations should not be understated. The specialist expertise of the operating surgeon appears to be important. Recent large studies report an increase in mortality in patients operated on by surgeons whose area of expertise was not in the area of the pathology, and for colorectal patients not operated on by specialist colorectal surgeons there were lower rates of laparoscopy and an increase in return to the operating room [30], although these results must be interpreted with caution as factors such as the urgency of the procedure may not have been fully accounted for. Another study shows lower mortality and stoma formation if an emergency colorectal resection is done by a colorectal subspecialist [31]. Solutions may include partnering across specialties in the management of complex emergency surgical patients [32, 33]. However, the authors acknowledge the availability of expertise will be dependent on the local health-care system.

### Recommendation

Perioperative care for patients undergoing emergency general surgery and specifically EL should be allocated to surgeons, anesthesiologists and intensivists with expertise that matches the needs of the patient. Strong consideration should be given as to how such resources are made available 24 h a day.

Level of evidence: High.

Recommendation grade: Strong.

## Ensuring the safe care of the emergency laparotomy patient postoperatively

### Postoperative levels of care

While some patients will not require advanced organ support, they may require enhanced or invasive monitoring and high staffing ratios, to prevent or rapidly manage complications [17, 34]. Availability of intensive care unit (ICU) beds may vary from country to country and system to system. Based on low numbers of ICU beds in the UK, national guidelines [35] initially stated that all patients with a risk score of ≥ 10% predicted mortality should be admitted directly to ICU postoperatively. This has now been reduced to a threshold of ≥ 5% predicted mortality [36] in updated guidelines, and includes all frail patients [37]. Using this revised threshold and a validated scoring system, planned admission to critical care for EL patients [38] has led to increased ICU admissions [36, 39]. A NELA report [36] showed that of 24,823 patients in 2020, 63% went directly to critical care (85% of those deemed high risk) and 5.4% were admitted to another “enhanced” area. For patients without a documented risk score, only 51.7% went directly to critical care. These findings were similar in a subsequent report [40]. Therefore, in the NELA data, patients who had their risk assessed formally before surgery were more likely to be admitted to critical care than high-risk patients who did not have a risk assessment. English NHS organizations are now incentivized to risk score and then admit patients with a predicted mortality of ≥ 5% directly to ICU by being paid through a best practice tariff.

In the InCare randomized clinical trial (RCT), provision of an intermediate postoperative level of care with continuous ECG monitoring, pulse oximetry and two hourly observations did not reduce mortality in EL patients [34]. However, the trial was stopped early due to less than expected mortality in both patient groups (7.6% intermediate care and 8.5% ward care 30-d mortality). In another study, extended stay in the postoperative anesthesia care unit (PACU) because of shortage of ICU beds increased mortality in critically ill emergency surgery patients [41]. Delayed admission to ICU following immediate postoperative discharge to a routine ward after surgery has been associated with increased mortality in larger studies [42, 43].

A pragmatic approach may be required for a risk score threshold triggering admission based on local availability of ICU beds. Patients who cannot be admitted to a critical care bed, require proactive and ongoing observation, and local protocols should be developed for this situation. The UK has published guidelines on the establishment of such units [44].

#### Recommendation

In an ideal health system, all patients would be admitted to ICU to have a high level of monitoring. A pragmatic approach may be required for a risk score threshold triggering admission based on local availability of ICU beds. Patients who cannot be admitted to a critical care bed require proactive and ongoing observation, and local protocols should be developed for this situation.

Level of evidence: Moderate.

Recommendation grade: Strong.

### Ongoing monitoring and management of ongoing physiological derangement, early detection of complications and avoidance of failure to rescue

Patients who have undergone EL remain at high risk of complications in the days following surgery. Proactive detection and management of physiological derangement and early management of complications need to continue well into the postoperative period [17, 45, 46]. A small prospective observational study [47] followed a cohort of 144 patients who had undergone EL every day for 28 days postoperatively and found the highest incidence of morbidity on day three. Preoperative American Society of Anesthesiologists (ASA) score and Portsmouth-Physiological and Operative Severity Score for the enUmeration of Mortality and morbidity (P-POSSUM) were significantly predictive of length of stay (LOS) and cumulative morbidity. Sixty percent of patients experienced at least one pulmonary, infectious or gastrointestinal complication, and complications were more common in patients over the age of 80 years. Although less common, cardiovascular and renal complications on any postoperative day were highly predictive of mortality, other major studies of non-cardiac surgery have found that cardiovascular complications are highly predictive of mortality [48, 49]. Acute kidney injury is also common in patients undergoing EL and predicts mortality [50, 51].

Track and trigger physiological scoring systems such as the early warning score alert to the development of a complication [52]. Early warning scores (EWS), prior to the incidence of the first major postoperative complication, were studied in 522 patients and predicted development of a complication and its severity up to 3 days prior to diagnosis of the complication [52]. A study of 6346 rapid response team (RRT) activations showed that the addition of EWS to other alerting criteria offered earlier detection for over half of the patients. However, use of EWS alone was not sensitive enough and required use of other criteria such as “worried” [53]. A retrospective study of 32,527 surgical inpatients found composite early warning scoring systems such as the electronic Cardiac Arrest Triage score (eCART), Modified EWS (MEWS) and National EWS (NEWS) to be highly predictive of a major adverse event [54]. eCART was slightly more accurate but has 33 variables and requires an electronic system, and MEWs and NEWs can be calculated from routine ward observation data. Selection of a risk score for a hospital or health-care system should be guided by available variables, calculation method, and system resources. Once implemented, high levels of adherence tied to specific levels of interventions, such as RRT activation, are necessary to allow the greatest potential to improve outcomes [55].

The failure to rescue (FTR) rate is the mortality rate for patients who experience complications. In one major study, the difference between high and low mortality hospitals was not determined by the incidence of the initial complication, but effective rescue of a patient once a complication had occurred, although this study covered a range of high-risk (high-risk defined as mortality >1%) general surgery and vascular surgery cases conditions, elective or emergency status was not specified [56]. FTR is modifiable by institutional factors such as higher nurse to patient ratios [57]. Retrospective studies have identified risk factors for FTR in EGS patients. A review of 329,183 patients [58] found a complication incidence of 21.2% in the 11,195 patients who died, with 64% experiencing more than one complication. Infectious and pulmonary complications were the commonest index complications and were synergistic in their potential to create a cascade of complications, which without effective rescue resulted in death. Another retrospective review of over 23,000 patients showed that risk adjusted FTR was significantly higher in older (≥75 years) patients after a first pulmonary or infectious complication, although not with a cardiovascular complication [59]. The FTR rate is higher in frail older populations [60, 61]. The risk of a specific pattern of secondary complications appears related to key index complications, for example deep space surgical site infection, is related to wound dehiscence [62]. Although the type of index complication is of note [59], it is action in the form of protocols to monitor, detect and act that reduce FTR and improve outcomes [57]. Rapid response teams which include an intensivist experienced in management of postoperative surgical patients have shown benefit [63]. Prevention of a cascade of events from initial complication to FTR provides a specific area for improvement in postoperative management of EL patients. Improvements in surgical mortality over the past 20 years for high-risk elective procedures in the Medicare database appear to have been achieved through improvements in FTR [64].

### Recommendation

Local protocols including use of a physiological track and trigger system to alert to deterioration and complication development should be developed, tailored to local service provision and infrastructure, to promote early intervention and prevent failure to rescue.

Level of evidence: High.

Recommendation grade: Strong.

#### Multidisciplinary management of the Emergency Laparotomy Patient

## Comprehensive proactive care of the older surgical patient

The impact of age and frailty on outcomes after EL was also discussed in Part 1 of these guidelines [1]. Age alone is significantly associated with poor outcomes for EL [27, 65], and many older patients are also frail, resulting in a lack of resilience in the face of a physiological insult [66–70]. Patients over 65 years of age should be screened for frailty using a validated tool [71]. Frailty is also strongly associated with an increased risk of delirium [72]. In a study of outcomes at 12 months in older patients after EL, the strongest predictors of mortality were frailty and increased ASA status [73]. A study using the NSQIP database showed a dose-dependent effect of frailty on FTR, as well as postoperative complications, reoperation, and all-cause mortality in older EGS patients [74]. An ERAS approach improved outcomes and reduced mortality in patients over the age of 70 years [14, 75, 76]. Although mortality is improving for older patients undergoing EGS, for those that survive large numbers do not return to their previous level of independence [77].

Involvement of a physician specialized in the care of older adults to co-manage these patients, and/or the use of targeted interventions should occur as soon as possible after surgery (if not before) [70]. Recent studies and guidelines provide evidence that proactive screening for frailty and comprehensive geriatric assessment (CGA) and management, improves outcomes in EGS [70, 78–81], and specifically for patients over 65 years of age who have undergone EL [15, 82]. One major study [81] used a proactive approach for EGS patients over 65 years including integration of a geriatric assessment team, promotion of patient-oriented rehabilitation, and early discharge planning, and found a significant reduction in mortality, LOS and discharge to a higher level of care [81]. Proactive management of frail patients may also decrease overall costs of care [83, 84]. At present, the evidence indicates that older EL patients are not reliably assessed for frailty nor co-managed with a care of the older-adult team [27].

### Recommendation

Patients over 65 years of age should be assessed, and co-managed, as early as possible postoperatively by a physician with expertise in the care of the older surgical patient (geriatrician) and evidence-based elder-friendly practices should be used.

Level of evidence: Moderate.

Recommendation grade: Strong.

## Implications of ERAS for emergency laparotomy for nursing practice

A key theme in successful implementation of ERAS pathways is the importance of the multidisciplinary team. Although the role of the nurse is always mentioned, there is little data examining the impact of the nursing role within an ERAS context, and of nursing care of the *emergency* patient group using an ERAS pathway [17]. Changes to nursing workload have been described in relation to elective ERAS [85], and nursing workload is likely to increase when applying ERAS to emergency patients [86]. Nursing care of this patient group is complicated by factors associated with EL such as increased frailty, pre-existing medical conditions [68] and cardiovascular concerns including orthostatic intolerance in addition to the lack of pre-habilitation due to the nature of presentation of these patients [17]. Nursing staff require a robust education program to ensure understanding of the rationale behind ERAS, including the need for early mobilization [87]. Nursing ratios decrease as the patient recovers and moves through different levels of postoperative care. One study [88] illustrated patients’ perspectives around step-down from critical care, interviews confirmed that the transition could be difficult, and patients felt insecure due to greater nursing workload and a perception of busier areas with fewer staff. In the UK specialist nursing roles focused on EL have evolved. NELA highlighted a need for continuity through transitions from critical care to general surgical areas, and for an outreach program [89].

Patients recovering from EL may experience issues with fatigue as well as pain [68] and consideration should be given to highlighting pathway goals such as mobilization targets [90]. To facilitate ERAS goal attainment multidisciplinary collaboration particularly between physiotherapy and nursing teams, and staff availability over weekends and out-of-hours, should be considered [17, 91]. Patients have reported [92] that weekends can be a challenging time during their recovery. Many emergency patients needing a stoma will not see a stoma nurse preoperatively and may go several days before seeing one postoperatively. The absence of preoperative stoma site marking, and emergency surgery have been identified as risk factors for development of a problematic stoma [93]. Much stoma specialist nursing is a weekday, office hours service.

### Recommendation

Nurses as well as other allied health professionals are key members of an ERAS team and should be involved in all stages of the design and implementation of the ERAS pathway.

Level of evidence: Low.

Recommendation grade*:* Strong.

Consideration should be given to the role of an EL program coordinator to facilitate data collection, promote ERAS pathway adherence and to provide a sense of continuity for patients as they progress through levels of care.

Level of evidence: Low.

Recommendation grade: Weak.

## End-of-life care and non-beneficial surgery

### Non-beneficial surgery and risk assessment

Up to one-third of older patients undergo surgery in the last year of life and 18% in the last month [94]. Most people do not wish to die in hospital and yet many will, often after invasive treatment such as surgery, which may be carried out at the expense of patient dignity [95]. Patients, families, and the multidisciplinary surgical team all wish to avoid non-beneficial surgery, a term which may be more acceptable than value-laden terms such as “futile,” or “inappropriate” [95]. The original definition of non-beneficial surgery was patients not surviving to hospital discharge after surgery; more recently, failure to survive to 48 h [96], 3 days [97] and 5 days [98] has been used. However, these definitions are binary (alive or dead) and overlook the principles of qualitative futility, which is of great importance for shared decision-making discussions between clinical teams and patients [99].

Risk assessment as a tool to help with discussion of risks and potential outcomes with patient and family, as well as multidisciplinary team (MDT) review before surgery for EL patients, and discussion about documentation of the patient’s goals of care preoperatively, are ERAS EL recommendations and were discussed in Part 1 of this guideline [1]. Discussions between specialties and disciplines with patients and caregivers regarding benefits and risks related to surgery, and the alternatives to surgery should be clearly documented as well as the patients’ “Goals of Care” [100]. Most risk prediction tools estimate mortality at 30-days on a population basis and should only be applied to an individual patient as part of an overall assessment. The NELA risk prediction tool is calibrated for EL and has better calibration for those at a higher risk of death than other common tools [101]. The Predictive Optimal Trees in Emergency Surgery Risk (POTTER) calculator is accurate and user-friendly in predicting 30-day mortality in patients undergoing EGS [102]. Although frailty is independently associated with increased perioperative mortality many earlier perioperative risk scores did not include frailty. A systematic review found that the NELA risk score combined with a frailty assessment and nutritional state gave a better prediction of mortality [103], where preoperative risk assessment predicts a particularly high-risk patient, and/or surgery is being considered in a patient with severe life-limiting disease and/or poor quality of life, English guidelines suggest shared decision-making should routinely include a senior surgeon, senior anesthetist, and senior intensivist as well as other senior specialists (e.g., geriatricians) if possible [37]. Formal risk assessment may not have occurred before surgery or findings at surgery may dramatically alter the likely outcome. Therefore, some guidelines suggest further risk assessment at the end of surgery [35, 37]. If not done prior to surgery, involvement of other health-care professionals such as a geriatrician or those with a longstanding relationship to the patient such as a primary care physician, should occur.

Postoperatively and in retrospect, there may be a situation where an operation is deemed non-beneficial. Discussion, at an MDT meeting to understand the reasons why the decision to operate was made, is likely to be helpful for surgical teams and future patients [104, 105]. Patients who did not have surgery, “NoLap” patients [106], should also be discussed at Morbidity and Mortality meetings. In addition to technical aspects, the conference should address surgical decision-making, multidisciplinary discussions, communication with the family and patient, and opportunities to improve patient and family experience, which may require input from end-of-life teams. To increase emotional support for trainees and help contextualize death in this difficult area, “end-of-life rounds” were developed for major abdominal surgical patients in the ICU [107, 108].

### The “NoLap” patient group

Until recently little has been known about patients who had indications for, but did not undergo, EL, termed the “NoLap” patient group. McIlveen et al. [106] studied patients eligible for laparotomy in a single center over ten months − 32% of those admitted (100 patients) with an acute intra-abdominal problem did not undergo EL and 63 died within 30-days. Poor documentation occurred in 16% of cases. The most common documented reasons for non-operative management were “poor fitness” and “not fit for surgery.” Another recent single-center study from Denmark reported contrasting results to McIlveen et al., with only 8.3% of eligible patients not undergoing surgery [109]. The 12-week prospective Perth Emergency Laparotomy Audit found that, of patients who died with an acute surgical abdomen, 43% were eligible for surgery but did not undergo EL [110]. More recently, in the Australia and New Zealand Emergency Laparotomy Audit-Quality Improvement pilot, 26% of patients’ eligible for surgery who died did not undergo laparotomy. While case ascertainment was low, this is likely to be an underestimate [111]. Traditionally non-operative management has been excluded from surgical quality performance assessments. Wandling et al. showed the ease of including non-operative care into surgical databases such as the ACS NSQIP to ensure quality is improved for all surgical patients, rather than focusing only on those who receive an operation [112]. Beginning in October 2022, NSQIP will include sampling of non-operative EGS patients, which will greatly strengthen the quality of research in this important EGS group.

There appears to be cultural differences in the willingness to offer operative management [109, 113, 114] and a paucity of evidence on what a good outcome means for patients, or medical professionals. A recent study tried to understand “recovery” for patients in four countries after abdominal surgery [115]. Returning to normal habits and routines, resolution of symptoms and regaining independence were overriding themes, demonstrating that for patients’ recovery goes beyond conventional parameters such as the absence of complications [115]. Understanding and collecting quality of life measurements for up to a year post-surgery will shape the care we deliver for these patients.

Future EL studies and databases should include, when possible, a study of patients who were eligible for surgery, but did not undergo surgery (the “NoLap” population) and died, to more fully represent the EGS population. Research should also explore international cultural differences in refusal for surgery.

There is a distinction between palliative care and care capped short of surgery, as they are not the same. A patient admitted for palliative care should ideally not be admitted under a surgeon and should not have any active surgical treatment, but rather comfort treatment and symptom management. A patient admitted for non-operative care may have very active treatment stopping short of surgery, and as McIlveen and colleagues showed [106] some of this group will leave hospital alive.

### Palliative care and end-of-life management

In the USA, almost two-thirds of older patients undergoing EL had baseline palliative care needs before surgery [116]. Poor communication among surgeons, patients and their advocates, and systemic factors may lead to non-beneficial procedures [117]. A common belief is that communication about end-of-life issues increases patient distress [117, 118]. However, specific interactions between physician and patient usually occur with a physician who does not know the patient, who may not address the patient’s wishes, and fails to provide enough information about prognosis to allow appropriate decisions [117, 118]. In addition, surgeons and anesthesiologists report a lack of confidence in talking to patients and their relatives about withdrawal of therapy, bereavement counseling, and palliative care [119]. Older patients undergoing emergency surgery often receive poor quality end-of-life care, leading to high rates of ICU admissions and in-hospital death, with low rates of hospice referral [118]. Poor communication is linked with adverse outcomes including increased physical and emotional suffering, prolonged care with little improvement in quality of life and poorer family satisfaction rates. When a patient undergoes non-beneficial treatment and the physician is aware but does not have the communication skills to prevent it, moral injury can occur [120]. The ACS has prioritized integration of palliative care principles in surgical training since 2003 [121] and the Royal College of Surgeons of England have produced a guide to good practice [122]. Ariadne Labs created the “Serious Illness Conversation Guide” [123] and supported a structured communication framework for older patients requiring emergency surgery [118]. Such resources can help support clinicians and provide guidance to better practice.

### Summary and recommendations

Future EL studies and databases should include, when possible, a study of patients who were eligible for surgery but did not undergo surgery (the “NoLap” population) and died. Research should also explore international cultural differences in refusal for surgery.

Level of evidence: Low.

Recommendation grade: Strong.

Multidisciplinary discussions should take place before surgery where feasible, for high-risk patients (high risk defined by local criteria using a validated risk scoring system including a frailty evaluation when appropriate) and after surgery on all eligible patients, as well as the “NoLap” population.

Level of Evidence; Moderate.

Recommendation; grade Strong.

Discussions between specialties and disciplines with patients and caregivers regarding benefits and risks related to surgery, and the alternatives to surgery should be clearly documented. The patients’ “Goals of Care” should be included in this documentation.

Level of Evidence: Low.

Recommendation grade: Strong.

Staff should have training in palliative care conversations and basic end-of-life management.

Level of Evidence: Low.

Recommendation grade: Weak.

## Audit, pathway implementation and improvement

Audit is a central component of ERAS [124]. Having a pathway with defined measurable components facilitates improvement and allows comparison of processes and outcomes between different practitioners and centers. Outcomes from EL and EGS have shown widespread variation ascribed to differences in patients, and variations in individual hospital performance [10, 125, 126]. There is a lack of data on the impact of patient triage for surgery, and relatively little data on variation of patient selection, and assessment of non-beneficial surgery [97, 106]. Variations in patients who undergo surgery can be accounted for to a certain extent with risk adjustment, but collection of all relevant data such as frailty and cognitive status is required. Variations in hospital performance are better addressed through quality improvement methodology.

An important component for improving care is mortality review which is a standard part of any audit and quality improvement program. Regular multidisciplinary meetings should be held, including not only surgeons and anesthesiologists but other relevant specialties such as radiologists, emergency department clinicians and geriatricians. Hospital executives should on occasion be involved, as any required system change may not be within the remit of clinicians [127]. Using a structured approach to review all laparotomy deaths can provide thematic insights into system, teamwork and communication issues and enable sharing and categorization of harm events, and development of areas for improvement. Such an approach to mortality review was mandated in the UK by the National Quality Board [128] and the use of standard nomenclature so that thematic analysis can be performed and lessons learnt across multiple specialties has also been promoted by the ACS [129].

**Fig. 2:**
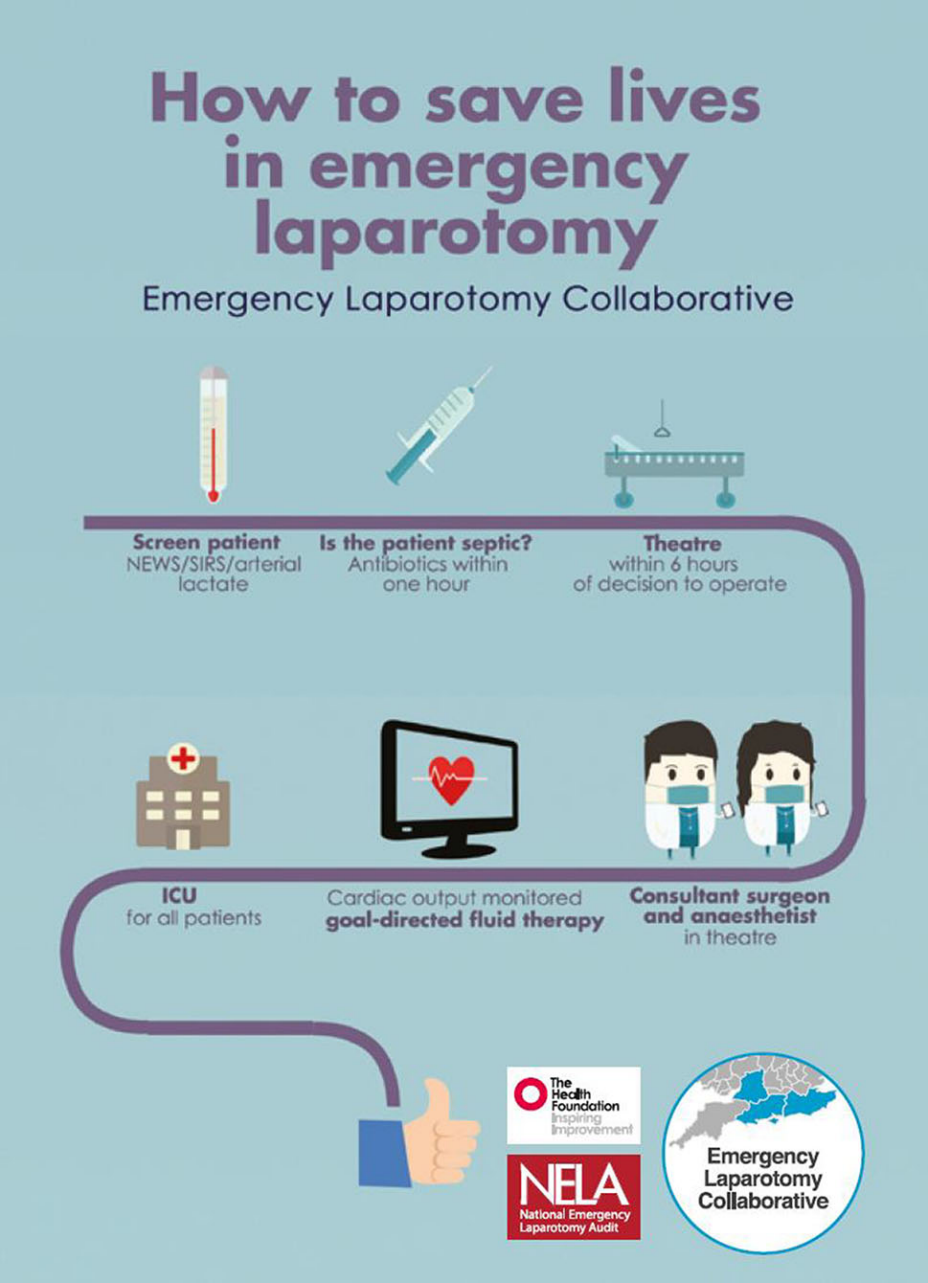
An illustrative example of an Emergency Laparotomy bundle, from Aggarwal et al. [14]

National data collection and reporting of performance of key metrics has been shown to be effective by NELA in the UK [130], and other countries have started similar projects [131, 132]. The way performance data are used, particularly timely and frequent feedback, has been associated with the effectiveness of the quality improvement program [133]. Attempts to implement too many ERAS components at once may be challenging, especially in the setting of emergency surgery, and when time to work on improvement is limited [134]. Approaching implementation of a smaller number of high impact components, such as in an EL care bundle or pathway [14, 45, 75, 135] may be more effective, especially when standard ERAS elements are already established (See Fig. 2 for an example of an ERAS pathway Emergency Laparotomy Bundle). One study showed that good compliance with a specific EL care bundle decreased mortality, but when compliance fell mortality increased [13]. When the bundle was re-applied with high compliance, mortality decreased again. Therefore, consideration of how improvements will be sustained should occur at the planning phase [13]. Increased use of quality improvement principles has been shown to aid improvement of process delivery for EL [136]. Guidance to help quality improvement and implementation is available [130, 137]. Changing care delivery is challenging, a protocol is not enough [138], real change needs not only clinical will and hard work, but cultural change and management support [13, 127, 134, 139]. Program success is associated with the adoption of evidence-based practices, assuring adequate resources are allocated for implementation and long-term sustainability.

### Recommendations

Audit is a central component of ERAS. Having a pathway with defined measurable components facilitates improvement and allows comparison of processes and outcomes between different practitioners and centers.

Level of evidence: Moderate.

Recommendation grade: Strong.

Outcomes from EL should be reviewed on a regular basis. Review should be multidisciplinary and multispecialty with performance compared against evidence-based standards. A structured approach with standard nomenclature and thematic analysis is essential and should be documented.

Level of evidence: Moderate.

Recommendation grade: Strong.

Development and implementation of an ERAS protocol for EL should involve patients and caregivers, multidisciplinary stakeholders, and senior executives. Adequate time and funding should be allocated for implementation and planning for long-term sustainability.

Level of evidence: Low.

Recommendation grade: Strong.

Lessons from large-scale studies suggest an implementation focus on a small number of high-impact components initially from an ERAS pathway may be more successful.

Level of evidence: Low.

Recommendation grade: Weak.

## Conclusions

These guidelines present a summary of the current evidence base and recommendations for key components of the organizational aspects of an ERAS pathway for EL. The quality of evidence is low in certain areas and much has been extrapolated from elective ERAS guidelines and elective intra-abdominal surgery; therefore, many components may require further prospective validation. We have addressed some components of care outside of the usual remit of an elective ERAS pathway, such as aspects of management of the patient who may be at their end-of-life, as we believe these issues must be addressed to provide a comprehensive and holistic patient-centered EL pathway. It is hoped that these organizational guidelines, paired with the preoperative and intraoperative guidelines, will provide a framework for improved management of patients undergoing EL. While there will be benefit in implementing certain components of the pathway, the greatest gain should be expected when organizational aspects of care are addressed, and the whole pathway is considered, implemented, and audited for compliance. Excitingly, the literature continues to grow in this area, and these guidelines will be reviewed and updated when appropriate.

## References

[1] Peden CJ, Aggarwal G, Aitken RJ (2021). Guidelines for perioperative care for emergency laparotomy enhanced recovery after surgery (ERAS) society recommendations: part 1-preoperative: diagnosis, rapid assessment and optimization. World J Surg.

[2] Guyatt GH, Oxman AD, Kunz R (2008). Going from evidence to recommendations. BMJ.

[3] Brown BB (1968). Delphi process: a methodology used for the elicitation of opinions of experts.

[4] Brindle M, Nelson G, Lobo DN (2020). Recommendations from the ERAS® Society for standards for the development of enhanced recovery after surgery guidelines. BJS Open.

[5] NELA Project Team (2017) Audit inclusion and exclusion criteria: NELA inclusion criteria. In: National emergency laparotomy audit. https://www.nela.org.uk/Criteria. Accessed 20 Aug 2019

[6] American College of Surgeons (2018) User guide for the 2017 ACS NSQIP participant use data file

[7] Havens JM, Peetz AB, Do WS (2015). The excess morbidity and mortality of emergency general surgery. J Trauma Acute Care Surg.

[8] Daniel VT, Ingraham AM, Khubchandani JA (2019). Variations in the delivery of emergency general surgery care in the era of acute care surgery. Jt Comm J Qual Patient Saf.

[9] Ingraham A, Nathens A, Peitzman A (2017). Assessment of emergency general surgery care based on formally developed quality indicators. Surgery.

[10] Ingraham AM, Cohen ME, Raval MV (2011). Comparison of hospital performance in emergency versus elective general surgery operations at 198 hospitals. J Am Coll Surg.

[11] Saunders DI, Murray D, Pichel AC (2012). Variations in mortality after emergency laparotomy: the first report of the UK Emergency Laparotomy Network. Br J Anaesth.

[12] Chana P, Joy M, Casey N (2017). Cohort analysis of outcomes in 69 490 emergency general surgical admissions across an international benchmarking collaborative. BMJ Open.

[13] Jordan LC, Cook TM, Cook S-C (2020). Sustaining better care for patients undergoing emergency laparotomy. Anaesthesia.

[14] Aggarwal G, Peden CJ, Mohammed MA (2019). Evaluation of the collaborative use of an evidence-based care bundle in emergency laparotomy. JAMA Surg.

[15] Oliver CM, Bassett MG, Poulton TE (2018). Organisational factors and mortality after an emergency laparotomy: multilevel analysis of 39 903 National emergency laparotomy audit patients. Br J Anaesth.

[16] Symons NRA, Moorthy K, Vincent CA, London Surgical Research Group (2016). Reliability in the process of care during emergency general surgical admission: a prospective cohort study. Int J Surg.

[17] Foss NB, Kehlet H (2020). Challenges in optimising recovery after emergency laparotomy. Anaesthesia.

[18] Coccolini F, Kluger Y, Ansaloni L (2018). WSES worldwide emergency general surgery formation and evaluation project. World J Emerg Surg.

[19] Association of Surgeons of Great Britain and Ireland and Royal College of Surgeons of England (2014) Commissioning guide: emergency general surgery (acute abdominal pain) https://www.rcseng.ac.uk/library-and-publications/rcs-publications/docs/emergency-general-guide/Accessed Jan 2023

[20] Royal College of Surgeons (2011) Emergency surgery: standards for unscheduled care. https://www.rcseng.ac.uk/library-and-publications/rcs-publications/docs/emergency-surgery-standards-for-unscheduled-care/

[21] American College of Surgeons (2022) Emergency general surgery verification program. https://www.facs.org/quality-programs/accreditation-and-verification/emergency-general-surgery/. Accessed 9 Jan 2023

[22] Mehta A, Dultz LA, Joseph B (2018). Emergency general surgery in geriatric patients: a statewide analysis of surgeon and hospital volume with outcomes. J Trauma Acute Care Surg.

[23] Ogola GO, Crandall ML, Richter KM, Shafi S (2018). High-volume hospitals are associated with lower mortality among high-risk emergency general surgery patients. J Trauma Acute Care Surg.

[24] To KB, Kamdar NS, Patil P (2019). Acute care surgery model and outcomes in emergency general surgery. J Am Coll Surg.

[25] Kasotakis G, Lakha A, Sarkar B (2014). Trainee participation is associated with adverse outcomes in emergency general surgery: an analysis of the National Surgical Quality Improvement Program database. Ann Surg.

[26] NELA Project Team (2018) Fourth patient report of the National Emergency Laparotomy Audit. RCoA London https://www.nela.org.uk/downloads/The%20Fourth%20Patient%20Report%20of%20the%20National%20Emergency%20Laparotomy%20Audit%202018%20-%20Full%20Patient%20Report.pdf Accessed Jan 2023

[27] NELA Project Team (2019) Fifth patient report of the National Emergency Laparotomy Audit https://www.nela.org.uk/downloads/The%20Fifth%20Patient%20Report%20of%20the%20NELA%202019%20-%20Full%20Patient%20Report.pdf Accessed Jan 2023

[28] Whitaker SR, Nisar S, Scally AJ, Radcliffe GS (2019). Does achieving the “Best Practice Tariff” criteria for fractured neck of femur patients improve one year outcomes?. Injury.

[29] Boyd-Carson H, Doleman B, Lockwood S (2020). Trainee-led emergency laparotomy operating. Br J Surg.

[30] Brown LR, McLean RC, Perren D (2019). Evaluating the effects of surgical subspecialisation on patient outcomes following emergency laparotomy: a retrospective cohort study. Int J Surg.

[31] Young J, Brown LR, Thomas CLG (2021). The impact of surgical subspecialization on patient outcomes following emergency colorectal resections in the north of England: a retrospective cohort study. Colorectal Dis.

[32] Boyd-Carson H, Doleman B, Herrod PJJ (2019). Association between surgeon special interest and mortality after emergency laparotomy. Br J Surg.

[33] Hallam S, Bickley M, Phelan L (2020). Does declared surgeon specialist interest influence the outcome of emergency laparotomy?. Ann R Coll Surg Engl.

[34] Vester-Andersen M, Waldau T, Wetterslev J (2015). Randomized multicentre feasibility trial of intermediate care versus standard ward care after emergency abdominal surgery (InCare trial). Br J Surg.

[35] The Royal College of Surgeons and Department of Health (2011) The higher risk general surgical patient: towards improved care for a forgotten group https://www.rcseng.ac.uk/library-and-publications/rcs-publications/docs/the-higher-risk-general-surgical-patient/ Accessed Jan 2023

[36] NELA Project Team (2020) Sixth patient report of the National Emergency Laparotomy Audit. https://www.hqip.org.uk/resource/national-emergency-laparotomy-audit-sixth-patient-report/. Accessed 20 Feb 2021

[37] Update to the high-risk surgical patient released by RCS England. https://www.nela.org.uk/RCS-Update-The-High-Risk-Surgical-Patient-Released. Accessed 20 Nov 2022

[38] Eugene N, Oliver CM, Bassett MG (2018). Development and internal validation of a novel risk adjustment model for adult patients undergoing emergency laparotomy surgery: the National Emergency Laparotomy Audit risk model. Br J Anaesth.

[39] STARSurg Collaborative, (2019). Critical care usage after major gastrointestinal and liver surgery: a prospective, multicentre observational study. Br J Anaesth.

[40] NELA Project Team (2021) Seventh patient report. https://www.nela.org.uk/Seventh-Patient-Report. Accessed 13 Mar 2022

[41] Bing-Hua YU (2014). Delayed admission to intensive care unit for critically surgical patients is associated with increased mortality. Am J Surg.

[42] Pearse RM, Moreno RP, Bauer P (2012). Mortality after surgery in Europe: a 7 day cohort study. Lancet.

[43] Gillies MA, Ghaffar S, Harrison E (2019). The association between ICU admission and emergency hospital readmission following emergency general surgery. Pediatr Crit Care Med.

[44] Mudford L Enhanced perioperative care. In: Centre for perioperative care. https://www.cpoc.org.uk/guidelines-resources-guidelines/enhanced-perioperative-care. Accessed 10 Jan 2023

[45] Tengberg LT, Bay-Nielsen M, Bisgaard T (2017). Multidisciplinary perioperative protocol in patients undergoing acute high-risk abdominal surgery. Br J Surg.

[46] Khuri SF, Henderson WG, DePalma RG (2005). Determinants of long-term survival after major surgery and the adverse effect of postoperative complications. Ann Surg.

[47] Howes TE, Cook TM, Corrigan LJ (2015). Postoperative morbidity survey, mortality and length of stay following emergency laparotomy. Anaesthesia.

[48] Devereaux PJ, Biccard BM, Writing Committee for the VISION Study Investigators (2017). Association of postoperative high-sensitivity troponin levels with myocardial injury and 30-day mortality among patients undergoing noncardiac surgery. JAMA.

[49] Zimmerman AM, Marwaha J, Nunez H (2016). Preoperative myocardial injury as a predictor of mortality in emergency general surgery: an analysis using the American College of Surgeons NSQIP Database. J Am Coll Surg.

[50] Briggs A, Havens JM, Salim A, Christopher KB (2018). Acute kidney injury predicts mortality in emergency general surgery patients. Am J Surg.

[51] Doyle JF, Sarnowski A, Saadat F (2019). Does the implementation of a quality improvement care bundle reduce the incidence of acute kidney injury in patients undergoing emergency laparotomy?. J Clin Med Res.

[52] Hollis RH, Graham LA, Lazenby JP (2016). A role for the early warning score in early identification of critical postoperative complications. Ann Surg.

[53] Reardon PM, Seely AJE, Fernando SM (2022). Can early warning systems enhance detection of high risk patients by rapid response teams?. J Intensive Care Med.

[54] Bartkowiak B, Snyder AM, Benjamin A (2019). Validating the electronic cardiac arrest risk triage (eCART) score for risk stratification of surgical inpatients in the postoperative setting: retrospective cohort study. Ann Surg.

[55] Churpek MM, Yuen TC, Edelson DP (2013). Risk stratification of hospitalized patients on the wards. Chest.

[56] Ghaferi AA, Birkmeyer JD, Dimick JB (2009). Variation in hospital mortality associated with inpatient surgery. N Engl J Med.

[57] Hatchimonji JS, Kaufman EJ, Sharoky CE (2019). Failure to rescue in surgical patients: a review for acute care surgeons. J Trauma Acute Care Surg.

[58] Hatchimonji JS, Swendiman RA, Kaufman EJ (2020). Multiple complications in emergency surgery: identifying risk factors for failure-to-rescue. Am Surg.

[59] Sheetz KH, Krell RW, Englesbe MJ (2014). The importance of the first complication: understanding failure to rescue after emergent surgery in the elderly. J Am Coll Surg.

[60] Shah R, Attwood K, Arya S (2018). Association of frailty with failure to rescue after low-risk and high-risk inpatient surgery. JAMA Surg.

[61] Khan M, Jehan F, Zeeshan M (2019). Failure to rescue after emergency general surgery in geriatric patients: does frailty matter?. J Surg Res.

[62] Wakeam E, Hyder JA, Jiang W (2015). Risk and patterns of secondary complications in surgical inpatients. JAMA Surg.

[63] Wakeam E, Asafu-Adjei D, Ashley SW (2014). The association of intensivists with failure-to-rescue rates in outlier hospitals: results of a national survey of intensive care unit organizational characteristics. J Crit Care.

[64] Fry BT, Smith ME, Thumma JR (2020). Ten-year trends in surgical mortality, complications, and failure to rescue in medicare beneficiaries. Ann Surg.

[65] Cooper Z, Mitchell SL, Gorges RJ (2015). Predictors of mortality up to 1 year after emergency major abdominal surgery in older adults. J Am Geriatr Soc.

[66] McIsaac DI, Moloo H, Bryson GL, van Walraven C (2017). The association of frailty with outcomes and resource use after emergency general surgery: a population-based cohort study. Anesth Analg.

[67] Akyar S, Armenia SJ, Ratnani P, Merchant AM (2018). The impact of frailty on postoperative cardiopulmonary complications in the emergency general surgery population. Surg J (N Y).

[68] Parmar KL, Law J, Carter B (2021). Frailty in older patients undergoing emergency laparotomy: results from the UK observational emergency laparotomy and frailty (ELF) study. Ann Surg.

[69] Tan HL, Chia STX, Nadkarni NV (2019). Frailty and functional decline after emergency abdominal surgery in the elderly: a prospective cohort study. World J Emerg Surg.

[70] Centre for Perioperative Care (2021) Perioperative care of people living with frailty. In: Centre for perioperative care. https://cpoc.org.uk/guidelines-resources-guidelines/perioperative-care-people-living-frailty. Accessed 14 Mar 2022

[71] Aucoin SD, Hao M, Sohi R (2020). Accuracy and feasibility of clinically applied frailty instruments before surgery: a systematic review and meta-analysis. Anesthesiology.

[72] Zhang X-M, Jiao J, Xie X-H, Wu X-J (2021). The association between frailty and delirium among hospitalized patients: an updated meta-analysis. J Am Med Dir Assoc.

[73] Vilches-Moraga A, Rowley M, Fox J (2020). Emergency laparotomy in the older patient: factors predictive of 12-month mortality—Salford-POPS-GS. An observational study. Aging Clin Exp Res.

[74] Kenawy DM, Renshaw SM, George E (2021). Increasing frailty in geriatric emergency general surgery: a cause for concern. J Surg Res.

[75] Huddart S, Peden CJ, Swart M (2015). Use of a pathway quality improvement care bundle to reduce mortality after emergency laparotomy. Br J Surg.

[76] Paduraru M, Ponchietti L, Casas IM (2017). Enhanced recovery after surgery (ERAS) - the evidence in geriatric emergency surgery: a systematic review. Chirurgia.

[77] Rubin DS, Huisingh-Scheetz M, Ferguson MK (2021). U.S. trends in elective and emergent major abdominal surgical procedures from 2002 to 2014 in older adults. J Am Geriatr Soc.

[78] Lee KC, Streid J, Sturgeon D (2020). The impact of frailty on long-term patient-oriented outcomes after emergency general surgery: a retrospective cohort study. J Am Geriatr Soc.

[79] Eamer G, Taheri A, Chen SS (2018). Comprehensive geriatric assessment for older people admitted to a surgical service. Cochrane Database Syst Rev.

[80] Engelhardt KE, Reuter Q, Liu J (2018). Frailty screening and a frailty pathway decrease length of stay, loss of independence, and 30-day readmission rates in frail geriatric trauma and emergency general surgery patients. J Trauma Acute Care Surg.

[81] Khadaroo RG, Warkentin LM, Wagg AS (2020). Clinical effectiveness of the elder-friendly approaches to the surgical environment initiative in emergency general surgery. JAMA Surg.

[82] Aitken RM, Partridge JSL, Oliver CM (2020). Older patients undergoing emergency laparotomy: observations from the National Emergency Laparotomy Audit (NELA) years 1–4. Age Ageing.

[83] Eamer GJ, Clement F, Holroyd-Leduc J (2019). Frailty predicts increased costs in emergent general surgery patients: a prospective cohort cost analysis. Surgery.

[84] Hofmeister M, Khadaroo RG, Holroyd-Leduc J (2020). Cost-effectiveness analysis of the elder-friendly approaches to the surgical environment (EASE) intervention for emergency abdominal surgical care of adults aged 65 years and older. JAMA Netw Open.

[85] Hübner M, Addor V, Slieker J (2015). The impact of an enhanced recovery pathway on nursing workload: a retrospective cohort study. Int J Surg.

[86] Lohsiriwat V, Jitmungngan R (2019). Enhanced recovery after surgery in emergency colorectal surgery: review of literature and current practices. World J Gastrointest Surg.

[87] Balfour A, Burch J, Fecher-Jones I, Carter FJ (2019). Understanding the benefits and implications of enhanced recovery after surgery. Nurs Stand.

[88] Thomsen T, Vester-Andersen M, Nielsen MV (2015). Patients' experiences of postoperative intermediate care and standard surgical ward care after emergency abdominal surgery: a qualitative sub-study of the Incare trial. J Clin Nurs.

[89] Stephenson J (2019) Nurses developing new roles to improve laparotomy care. In: Nursing times. https://www.nursingtimes.net/news/research-and-innovation/nurses-developing-new-roles-to-improve-laparotomy-care/7028566.article. Accessed 31 Mar 2021

[90] Poulton T, Murray D, National Emergency Laparotomy Audit (NELA) project team (2019). Pre-optimisation of patients undergoing emergency laparotomy: a review of best practice. Anaesthesia.

[91] Boden I, Sullivan K, Hackett C (2018). ICEAGE (Incidence of Complications following Emergency Abdominal surgery: Get Exercising): study protocol of a pragmatic, multicentre, randomised controlled trial testing physiotherapy for the prevention of complications and improved physical recovery after emergency abdominal surgery. World J Emerg Surg.

[92] Law J, Welch C, Javanmard-Emamghissi H (2020). Decision-making for older patients undergoing emergency laparotomy: defining patient and clinician values and priorities. Colorectal Dis.

[93] Parmar KL, Zammit M, Smith A (2011). A prospective audit of early stoma complications in colorectal cancer treatment throughout the Greater Manchester and Cheshire colorectal cancer network. Colorectal Dis.

[94] Kwok AC, Semel ME, Lipsitz SR (2011). The intensity and variation of surgical care at the end of life: a retrospective cohort study. Lancet.

[95] Cardona-Morrell M, Kim J, Turner RM (2016). Non-beneficial treatments in hospital at the end of life: a systematic review on extent of the problem. Int J Qual Health Care.

[96] Chiu AS, Jean RA, Resio B, Pei KY (2019). Early postoperative death in extreme-risk patients: a perspective on surgical futility. Surgery.

[97] Aggarwal G, Broughton KJ, Williams LJ (2020). Early postoperative death in patients undergoing emergency high-risk surgery: towards a better understanding of patients for whom surgery may not be beneficial. J Clin Med.

[98] DeWane MP, Davis KA, Schuster KM (2019). Rethinking our definition of operative success: predicting early mortality after emergency general surgery colon resection. Trauma Surg Acute Care Open.

[99] Javanmard-Emamghissi H, Lockwood S, Hare S (2022). The false dichotomy of surgical futility in the emergency laparotomy setting: scoping review. BJS Open.

[100] Roberts GP, Levy N, Lobo DN (2021). Patient-centric goal-oriented perioperative care. Br J Anaesth.

[101] Eliezer DD, Holmes M, Sullivan G (2020). High-risk emergency laparotomy in Australia: comparing NELA, P-POSSUM, and ACS-NSQIP calculators. J Surg Res.

[102] El Hechi MW, Maurer LR, Levine J (2021). Validation of the artificial intelligence-based predictive optimal trees in emergency surgery risk (POTTER) calculator in emergency general surgery and emergency laparotomy patients. J Am Coll Surg.

[103] Barazanchi A, Bhat S, Palmer-Neels K (2020). Evaluating and improving current risk prediction tools in emergency laparotomy. J Trauma Acute Care Surg.

[104] Foss NB (2020). Emergency laparotomy success – optimisation or triage?. Anaesthesia.

[105] Sroka R, Gabriel EM, Al-Hadidi D (2019). A novel anesthesiologist-led multidisciplinary model for evaluating high-risk surgical patients at a comprehensive cancer center. J Healthc Risk Manag.

[106] McIlveen EC, Wright E, Shaw M (2020). A prospective cohort study characterising patients declined emergency laparotomy: survival in the “NoLap” population. Anaesthesia.

[107] Cooper Z, Lilley EJ, Bollens-Lund E (2018). High burden of palliative care needs of older adults during emergency major abdominal surgery. J Am Geriatr Soc.

[108] Khot S, Billings M, Owens D, Longstreth WT (2011). Coping with death and dying on a neurology inpatient service: death rounds as an educational initiative for residents. Arch Neurol.

[109] Ebrahim M, Lauritsen ML, Cihoric M (2023). Triage and outcomes for a whole cohort of patients presenting for major emergency abdominal surgery including the No-LAP population: a prospective single-center observational study. Eur J Trauma Emerg Surg.

[110] Broughton KJ, Aldridge O, Pradhan S, Aitken RJ (2017). The Perth emergency laparotomy audit. ANZ J Surg.

[111] Aitken RJ, Griffiths B, The ANZELA-QI Working Party (2021). Two-year outcomes from the Australian and New Zealand emergency laparotomy audit-quality improvement pilot study. ANZ J Surg.

[112] Wandling MW, Ko CY, Bankey PE (2017). Expanding the scope of quality measurement in surgery to include nonoperative care: results from the American College of Surgeons National Surgical Quality Improvement Program emergency general surgery pilot. J Trauma Acute Care Surg.

[113] Markar SR, Vidal-Diez A, Patel K (2019). Comparison of surgical intervention and mortality for seven surgical emergencies in England and the United States. Ann Surg.

[114] Markar SR, Vidal-Diez A, Holt PJ (2021). An international comparison of the management of gastrointestinal surgical emergencies in Octogenarians-England versus United States: a national population-based cohort study. Ann Surg.

[115] Rajabiyazdi F, Alam R, Pal A (2021). Understanding the meaning of recovery to patients undergoing abdominal surgery. JAMA Surg.

[116] Lilley EJ, Cooper Z (2016). The high burden of palliative care needs among older emergency general surgery patients. J Palliat Med.

[117] Cooper Z, Courtwright A, Karlage A (2014). Pitfalls in communication that lead to nonbeneficial emergency surgery in elderly patients with serious illness: description of the problem and elements of a solution. Ann Surg.

[118] Cooper Z, Koritsanszky LA, Cauley CE (2016). Recommendations for best communication practices to facilitate goal-concordant care for seriously ill older patients with emergency surgical conditions. Ann Surg.

[119] Bakke KE, Miranda SP, Castillo-Angeles M (2018). Training surgeons and anesthesiologists to facilitate end-of-life conversations with patients and families: a systematic review of existing educational models. J Surg Educ.

[120] Dean W, Talbot S, Dean A (2019). Reframing clinician distress: moral injury not burnout. Fed Pract.

[121] Surgeons Palliative Care Workgroup (2003). Office of promoting excellence in end-of-life care: surgeon's palliative care workgroup report from the field. J Am Coll Surg.

[122] sitecore\ Caring for patients nearing the end of life. In: Royal College of Surgeons. https://www.rcseng.ac.uk/standards-and-research/standards-and-guidance/good-practice-guides/end-of-life-care/. Accessed 6 Feb 2022

[123] Bernacki RE, Block SD, American College of Physicians High Value Care Task Force (2014). Communication about serious illness care goals: a review and synthesis of best practices. JAMA Intern Med.

[124] Francis NK, Walker T, Carter F (2018). Consensus on training and implementation of enhanced recovery after surgery: a Delphi study. World J Surg.

[125] Columbus AB, Morris MA, Lilley EJ (2018). Critical differences between elective and emergency surgery: identifying domains for quality improvement in emergency general surgery. Surgery.

[126] Ingraham AM, Ayturk MD, Kiefe CI, Santry HP (2019). Adherence to 20 emergency general surgery best practices: results of a national survey. Ann Surg.

[127] Stephens TJ, Beckingham IJ, Bamber JR, Peden CJ (2022). What influences the effectiveness of quality improvement in perioperative care: learning from large multicenter studies in emergency general surgery?. Anesth Analg.

[128] (2018) Mortality toolkit: Implementing structured judgement reviews for improvement. In: RCP London. https://www.rcplondon.ac.uk/guidelines-policy/mortality-toolkit-implementing-structured-judgement-reviews-improvement. Accessed 10 Feb 2022

[129] American College of Surgeons. Committee on Trauma (1990) Resources for optimal care of the injured patient. American College of Surgeons, Committee on Trauma

[130] The National Emergency Laparotomy Audit (NELA). https://www.nela.org.uk/. Accessed 20 Jun 2019

[131] ANZ Emergency Laparotomy Audit – Quality improvement. https://www.surgeons.org/research-audit/morbidity-audits/morbidity-audits-managed-by-racs/anz-emergency-laparotomy-audit-quality-improvement. Accessed 20 Jun 2020

[132] Liljendahl MS, Gögenur I, Thygesen LC (2020). Emergency laparotomy in Denmark: a nationwide descriptive study. World J Surg.

[133] Campbell DA, Englesbe MJ, Kubus JJ (2010). Accelerating the pace of surgical quality improvement: the power of hospital collaboration. Arch Surg.

[134] Peden CJ, Stephens T, Martin G (2019). Effectiveness of a national quality improvement programme to improve survival after emergency abdominal surgery (EPOCH): a stepped-wedge cluster-randomised trial. Lancet.

[135] Møller MH, Adamsen S, Thomsen RW (2011). Multicentre trial of a perioperative protocol to reduce mortality in patients with peptic ulcer perforation. Br J Surg.

[136] Stephens TJ, Peden CJ, Haines R (2019). Hospital-level evaluation of the effect of a national quality improvement programme: time-series analysis of registry data. BMJ Qual Saf.

[137] Institute for Healthcare Improvement How to Improve. http://www.ihi.org/resources/Pages/HowtoImprove/default.aspx. Accessed 5 Jul 2018

[138] Maessen J, Dejong CHC, Hausel J (2007). A protocol is not enough to implement an enhanced recovery programme for colorectal resection. Br J Surg.

[139] Stephens TJ, Peden CJ, Pearse RM (2018). Improving care at scale: process evaluation of a multi-component quality improvement intervention to reduce mortality after emergency abdominal surgery (EPOCH trial). Implement Sci.

